# p97 Inhibition Synergistically Enhances Hypomethylating Therapy through Targeting of PLK1 in Acute Myeloid Leukemia

**DOI:** 10.1158/2767-9764.CRC-26-0035

**Published:** 2026-06-26

**Authors:** Steffan T. Nawrocki, Ning Wang, Claudia M. Espitia, Harley Wu, Claudia Villa Celi, Julian Olea, Paniz Zarand, Homa Dadrastoussi, Hittu Matta, Sruthi Sureshkumar, Madison E. Gamble, Natalie L. Hakim, David Drum, Jack Angeles, Kaijin Wu, Jiang F. Zhong, Xuelian Chen, Alex Duong, Jennifer S. Carew, Kevin R. Kelly

**Affiliations:** 1 Division of Translational Medicine, Department of Medicine, University of Arizona Cancer Center, Tucson, Arizona.; 2 Division of Hematology, Department of Medicine, University of Southern California, Los Angeles, California.; 3 Division of Hematology, Health Sciences Campus, University of Southern California, Los Angeles, California.; 4 Division of Hematology and Oncology, Cleveland Clinic Florida, Weston, Florida.; 5 Department of Basic Science, School of Medicine, Loma Linda University, Loma Linda, California.

## Abstract

Acute myeloid leukemia (AML) remains defined by therapeutic resistance, adverse genetic heterogeneity, and poor long-term survival, underscoring the need for mechanistically informed regimens that exploit core stress-adaptation liabilities. Polo-like kinase 1 (PLK1) is a central regulator of replication recovery and mitotic progression, yet direct PLK1 inhibitors have been limited by tolerability and incomplete target suppression. Here, we define a clinically actionable strategy that functionally targets PLK1 by combining inhibition of the AAA+ ATPase p97/valosin-containing protein with the hypomethylating agent decitabine (DAC). Using AML cell lines, primary patient specimens, and an orthotopic *in vivo* model, we show that the clinically relevant p97 inhibitor CB-5339 induces proteotoxic and replication stress, activates the unfolded protein response, and triggers apoptosis. Formal synergy analyses demonstrate that CB-5339 and DAC cooperate across molecular subtypes, including *FLT3-*ITD+, *KMT2A*-rearranged, and *TP53*-mutant AML, to reduce cell viability at pharmacologically relevant concentrations. Transcriptomic, biochemical, and genetic studies identify synergistic suppression of PLK1 as a central consequence of combination treatment, converting stress-dependent PLK1 reliance into a therapeutic vulnerability. Consistent with this mechanism, PLK1 knockdown phenocopies the antileukemic effects of the combination and enhances sensitivity to both agents. *In vivo*, CB-5339/DAC is well tolerated, significantly prolongs survival, reduces leukemic burden, and suppresses PLK1 in bone marrow blasts. Together, these data establish p97 inhibition as a rational means to exploit replication and proteotoxic stress in AML and provide strong rationale for clinical evaluation of CB-5339 plus DAC in high-risk disease.

**Significance:**

This study defines a clinically actionable strategy to overcome therapeutic resistance in AML by functionally suppressing PLK1 through combined p97 inhibition and hypomethylating therapy. By exploiting convergent proteotoxic and replication stress, the regimen produces robust, well-tolerated antileukemic activity across high-risk genetic subtypes, supporting near-term clinical evaluation.

## Introduction

Despite recent advances in molecular profiling and risk stratification, the therapeutic landscape for acute myeloid leukemia (AML) remains constrained by intrinsic and acquired resistance to standard-of-care regimens. Hypomethylating agents such as decitabine (DAC) and azacitidine remain foundational therapies for older patients and those unfit for intensive induction, yet durable remissions are uncommon, and relapse is nearly universal (1). Resistance to hypomethylating therapy reflects AML’s capacity to adapt to sustained genotoxic, epigenetic, and metabolic stress through coordinated activation of DNA damage response pathways, cell-cycle checkpoints, and proteostasis networks (2–4). Together, these adaptive programs enable leukemic cells to tolerate chronic replication stress and evade apoptosis, highlighting the need for new strategies that disable stress resolution rather than targeting individual oncogenic drivers.

Proteostasis has emerged as a critical vulnerability in AML, a disease marked by high protein turnover, elevated basal stress signaling, and reliance on efficient protein quality control mechanisms (5, 6). The AAA+ ATPase p97/valosin-containing protein plays a central role in these processes, functioning as a segregase that extracts ubiquitinated substrates from protein complexes, chromatin, and organellar membranes for downstream processing or degradation (7). Through these activities, p97 supports endoplasmic reticulum (ER)-associated degradation, DNA damage repair, replication fork recovery, and cell-cycle progression (8). AML cells seem particularly dependent on p97-mediated stress adaptation, positioning p97 as an attractive therapeutic target capable of disrupting multiple survival pathways simultaneously (9).

p97 also operates at the intersection of proteotoxic stress and genome maintenance, placing it squarely within the cellular response to hypomethylating therapy. DAC induces replication stress through DNA incorporation and DNMT1 depletion, leading to stalled replication forks and DNA lesions that must be resolved to preserve viability (10). In addition, another key mechanism of action of DAC is induction of mitotic disruption (11). p97 contributes to the clearance of stalled replication machinery and facilitates recovery from replication-associated damage, suggesting that p97 inhibition may selectively compromise AML cells exposed to hypomethylating agents (12). This convergence enables p97 blockade to convert the largely cytostatic effects of DAC into a cytotoxic response by preventing adaptive stress resolution, exposing a broader vulnerability in AML stress tolerance.

A recent study identified a link between p97 and Polo-like kinase 1 (PLK1), a central regulator of mitotic progression and the DNA damage response (13). PLK1 is frequently overexpressed in AML and is consistently associated with adverse disease biology, therapeutic resistance, and inferior survival (14–17). Despite compelling preclinical rationale, direct pharmacologic inhibition of PLK1 has failed to translate into durable clinical benefit, exemplified by the limited efficacy and tolerability of the PLK1 inhibitor volasertib in late-stage trials (18). These outcomes highlight a fundamental limitation of targeting essential mitotic kinases in AML as sustained inhibition of PLK1 is poorly tolerated, yet incomplete inhibition is therapeutically ineffective. An alternative strategy is to functionally suppress PLK1 by destabilizing the cellular conditions that sustain its expression and activity. Indirect targeting approaches that reduce PLK1 abundance or disrupt its upstream regulatory networks may achieve therapeutic benefit while avoiding the liabilities associated with direct kinase inhibition. Given p97’s role in proteostasis and transcriptional regulation under stress conditions, p97 inhibition represents a compelling mechanism to indirectly impair PLK1 function, particularly when combined with the replication stress imposed by hypomethylating therapy.

In this study, we define the therapeutic potential of combining the clinically relevant second-generation p97 inhibitor CB-5339 with DAC in AML. We show that this combination exploits convergent proteotoxic and replication stress pathways to produce synergistic antileukemic activity across genetically diverse and high-risk AML subtypes. Mechanistic studies identify PLK1 as a central pharmacodynamic target of the combination, linking stress overload to collapse of a critical cell-cycle survival axis. Collectively, these findings establish a mechanistically informed framework for leveraging p97 inhibition to functionally target PLK1 and overcome resistance to hypomethylating therapy in AML.

## Materials and Methods

### Ethics approval and patient consent to participate

This study was conducted at the University of Southern California and the University of Arizona. Primary AML samples from the bone marrow of patients at the University of Southern California were obtaining after written informed consent in accordance with an approved Institutional Review Board (IRB) protocol (IRB No. HS-10-0051). The study was conducted in accordance with Good Clinical Practice Guidelines and the Declaration of Helsinki.

### AML cells and cell culture

The AML cell lines MOLM-13, PL-21, SKM-1, and NOMO-1 were obtained from DSMZ. MV4-11, KG-1, and HL-60 were purchased from the American Type Culture Collection. Cell lines were validated using short tandem repeat DNA profiling. All cells are maintained in a *Mycoplasma*-free environment. Normal human CD34^+^ bone marrow cells were obtained from Stem Cell Technologies. Cells were cultured in Roswell Park Memorial Institute (RPMI) 1640 medium containing 10% fetal bovine serum (FBS) and 1% penicillin–streptomycin. Cells were cultured in a humidified atmosphere containing 5% CO_2_ at 37°C.

### Antibodies and reagents

Primary antibodies were obtained from Cell Signaling Technology for the following proteins: K48 ubiquitin (cat. #8081; RRID: AB_10859893), γ-H2AX (cat. #9718; RRID: AB_2118009), poly (ADP-ribose) polymerase (PARP; cat. #9542; RRID: AB_2160739), spliced X-box binding protein 1 (XBP1s; cat. #12782; RRID: AB_2687943), C/EBP homologous protein (CHOP; cat. #2895; RRID: AB_2089254), activating transcription factor 6 (ATF-6; cat. #65880; RRID: AB_2799696), and β-actin (cat. #3700; RRID: AB_2242334). Primary antibody against glyceraldehyde 3-phosphate dehydrogenase (GAPDH; cat. #60004-1-Ig; RRID: AB_2107436) was obtained from Proteintech. Primary antibody against caspase-3 was obtained from Thermo Fisher Scientific (cat. #MA1-91637; RRID: AB_1954999). Primary antibody against PLK1 (cat. #NBP3-32829; RRID: AB_2932356) was obtained from Novus Biologicals. Horseradish peroxidase (HRP)-conjugated secondary antibodies (cat. #sc-2318; RRID: AB_641171; cat. #sc-2313; RRID: AB_641181; cat. #sc-2357; RRID: AB_628497) and HRP-conjugated mouse IgG BP (cat. #sc-52548; RRID: AB_629966; cat. #sc-52549; RRID: AB_629945) were obtained from Santa Cruz Biotechnology. 3-(4,5-Dimethylthiazol-2-yl)-2,5-diphenyl-2H-tetrazolium bromide (MTT) was obtained from Sigma-Aldrich (cat. #M2128). DAC and venetoclax were acquired from MedChemExpress (cat. #HY-A0004). CB-5339 was provided by Cleave Therapeutics (now Casi Pharmaceuticals).

### Immunoblotting

To assess protein expression levels, 2 to 5 × 10^6^ cells were collected for each condition and analyzed by immunoblotting. First, cells were rinsed twice with phosphate-buffered saline (PBS), followed by the addition of radioimmunoprecipitation assay (RIPA) buffer [50 mmol/L Tris-HCl, pH 7.5; 150 mmol/L NaCl; 1% NP-40; 0.5% sodium deoxycholate; and 0.1% sodium dodecyl sulfate (SDS)] containing a protease inhibitor cocktail. Cells were lysed on ice for 30 minutes and centrifuged at 12,000 × *g* for 10 minutes at 4°C. Supernatants were collected, and protein concentrations were measured using Pierce BCA Protein Assay Kit. After denaturation at 96°C for 5 minutes, 20 μg of each protein sample was subjected to 4% to 12% SDS–polyacrylamide gel electrophoresis and transferred to a nitrocellulose membrane. The membrane was blocked with 5% Blotting-Grade Blocker (Bio-Rad) in Tris-buffered saline containing 0.1% Tween 20 (TBST) for 1 hour, followed by overnight incubation at 4°C with primary antibodies. Membranes were washed three times for 5 minutes each, followed by incubation with goat polyclonal anti-mouse or anti-rabbit HRP-conjugated secondary antibodies in 5% Blotting-Grade Blocker for 1 hour. After washing with TBST three times for 5 minutes each, the membrane was incubated with SuperSignal West Femto Maximum Sensitivity Substrate (Thermo Fisher Scientific, cat. #34095). The signals were developed using the Gel Doc XR+ Gel Documentation System (Bio-Rad). The images and the intensities of bands were obtained using Image Lab software (Bio-Rad; RRID: SCR_014210). Quantification of the intensity of immunoblotting bands was performed using ImageJ software.

### K48 ubiquitination immunoblotting

Primary AML cells were obtained from three patients for immunoblotting. Cells (2 × 10^7^) were treated in RPMI media with 50, 150, or 300 nmol/L CB-5339 and harvested at the indicated time points. Cells were lysed in RIPA buffer, and 20 μg protein was separated on 4% to 12% NuPAGE Bis-Tris minigels and transferred to nitrocellulose membrane. After blocking with 5% Bio-Rad blocker, membranes were incubated with rabbit anti-K48 monoclonal antibody for 2 hours, incubated with HRP-tagged secondary goat anti-rabbit antibody, and visualized with enhanced chemiluminescence reagents (Thermo Fisher Scientific, SuperSignal West Femto Maximum Sensitivity Substrate, cat. #34095).

### Quantification of drug-induced cytotoxicity

Cell viability was assessed by MTT assay. Cells were seeded into 96-well microplates at 5,000 cells per well. Cells were then treated with CB-5339, DAC, and combinations for 72 hours. Following drug treatment, MTT was added, and cell viability was quantified using a Molecular Devices microplate reader. Proapoptotic effects following *in vitro* drug exposure were quantified by propidium iodide (PI) staining and fluorescence-activated cell sorting (FACS) analysis of sub-G_0_/G_1_ DNA content as previously described (19).

### Synergy analysis

The combination indices (CI) for CB-5339 and DAC were calculated using 72-hour MTT assays (20). CompuSyn software (ComboSyn) was utilized to calculate CI values. In addition, synergy scores were determined using the highest single agent method as previously described (21).

### Annexin-V flow cytometry

AML cells were treated simultaneously with CB-5339 (MOLM-13, 300 nmol/L; MV4-11, and 200 nmol/L) and 1 μmol/L DAC for 48 hours, stained using FITC Annexin-V Apoptosis Detection Kit (BD Pharmingen; cat. #556547), and subjected to flow cytometry. The proportion of apoptotic cells was quantified using a BD FACSVerse flow cytometer.

### RNA sequencing

MOLM-13 and MV4-11 cells were grown to a cell density of 1 million cells/mL. For DAC experiments, cells were treated with 1 μmol/L DAC for 24 hours. After 24 hours, the culture medium was replaced with medium containing 1 μmol/L DAC or 1 μmol/L DAC + 500 nmol/L CB-5339 for 6, 12, or 18 hours. For control (DMSO) and CB-5339–only experiments, cells were treated for 6, 12, or 18 hours. Each condition used three biological replicates. Total RNA was prepared from approximately 6 million cells per condition using QIAGEN RNeasy Plus Mini Kit (cat. #74134). RNA library construction and sequencing were performed by Novogene using a NovaSeq 6000 S4 flow cell and sequenced on Illumina platforms at a depth of 9 Gb. The raw data for each experimental condition were processed through Partek Flow. The reads were trimmed from both ends and then aligned using STAR. The data were normalized via counts per million, and gene set analysis was conducted. Extracted gene sets were imported into Ingenuity Pathway Analysis (RRID: SCR_008653) to filter canonical pathways, disease-related genes, and functions. The FDR *P* value was >0.05. The top 20 upregulated and downregulated genes across both AML cell line models were determined. PLK1 was selected for further investigation based on its consistent downregulation following CB-5339 + DAC treatment and its previously reported key role in AML (22, 23).

### Quantitative real-time polymerase chain reaction

cDNA from AML cells were used for relative quantification by real-time polymerase chain reaction (qRT-PCR) analyses. First-strand cDNA synthesis was performed from 1 μg RNA in a 20 μL reaction mixture using the high-capacity cDNA Reverse Transcription Kit (Applied Biosystems). *PLK1* and *18S* rRNA cDNAs were amplified using commercially available TaqMan Gene expression assays (Applied Biosystems).

### shRNA silencing of *PLK1*

In accordance with the manufacturer’s guidelines (Santa Cruz Biotechnology), MOLM-13 cells were transduced with lentiviral particles containing pool nontargeted (control; cat. #sc-108080) or target-specific short hairpin RNA (shRNA) directed at *PLK1* (cat. #sc-36277-V). Effective transduction was selected for with puromycin. Transduced cells were treated with the designated drugs and concentrations. Immunoblotting was used to determine PLK1 knockdown efficiency.

### Overexpression of *PLK1*

MOLM-13 cells were infected with lentiviral ORF control particles of pLenti-C-Myc-DDK-P2A-Puro (cat. #PS100092V) or lentiviral *PLK1* particles of pLenti-C-Myc-DDK-P2A-Puro (cat. #RC201795L3V) according to the manufacturer’s protocol (OriGene). Successful transfection was selected for with puromycin. Cell viability was assessed by MTT assay. Overexpression efficiency was confirmed by immunoblotting.

### *In vivo* evaluation of CB-5339 and DAC

The animal experiments were performed with the approval of the Institutional Animal Care and Use Committee of the University of Arizona (16-094) and were conducted in accordance with established animal welfare guidelines. MV4-11 cells (0.5 × 10^6^) were suspended in Hank’s Balanced Salt Solution and injected into the tail veins of NOD.Cg-*Prkdc*^*scid*^*Il2rg*^*tm1Sug*^/JicTac (NOG) mice (RRID: IMSR_TAC:HSCFTL-NOG; Taconic). Leukemic mice were randomized into treatment groups and treated with vehicle, 50 mg/kg CB-5339 (orally administered daily on days 1–5), 2.5 mg/kg DAC (injected intraperitoneally on days 1, 3, and 5), or both drugs as indicated. Treatment cycles were 7 days and were repeated until study end. Mice were monitored daily, and overall survival was quantified. Animal weight was measured twice a week. At study completion, bone marrow specimens from representative animals were excised from each group, formalin-fixed, and paraffin-embedded for immunohistochemical (IHC) analysis.

### IHC

Paraffin-embedded tumor sections were deparaffinized in xylene, exposed to a graded series of alcohol, and rehydrated in PBS (pH 7.5). Heat-induced epitope retrieval on the sections and probing with specific antibodies was conducted as previously described (24). ImageJ software was used for quantification of PLK1 levels by densitometric analysis of five random fields containing viable tumor cells (24). Quantification of CD33^+^ cells was conducted by counting the number of positive cells in five random fields (25).

### Analysis of BeatAML2 datasets

The BeatAML2 datasets (Integrative Analysis of Drug Response and Clinical Outcome in Acute Myeloid Leukemia|BeatAML2), which include gene expression, clinical annotation, and drug response data, were analyzed using R v4.5.0. The ELN2022 risk stratification was used to categorize patients into favorable- and adverse-risk groups for further analysis. Normalized transcriptomic data were used to extract *PLK1* gene expression values. Drug response to hypomethylating therapy (azacitidine) was quantified using the area under the curve (AUC; ref. 26). Higher AUC values indicate increased resistance. Comparisons between the two groups were conducted using the two-sided Wilcoxon rank-sum test.

### Statistical analyses

Statistical significance of differences among samples were determined using the Student *t* test and one-way ANOVA analysis where appropriate. Survival curves were determined by Kaplan–Meier analysis and compared using the log-rank test. Differences were considered significant in all experiments at *P* < 0.05.

## Results

### Inhibition of p97 reduces AML cell viability

**Figure 1.**
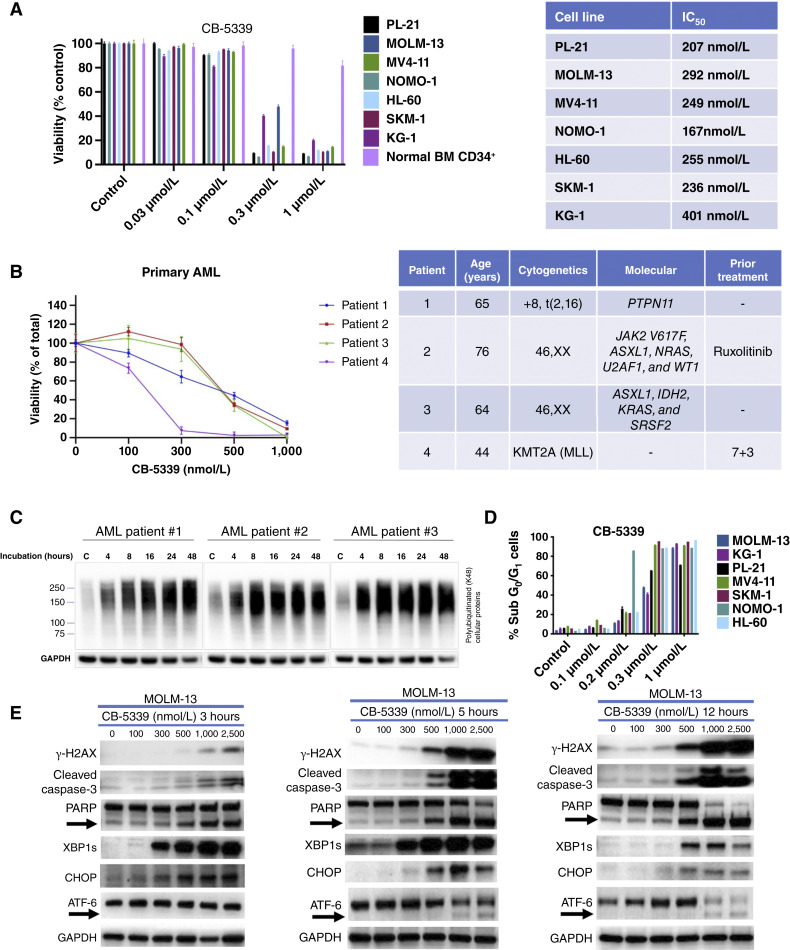
CB-5339 reduces AML cell viability in cell lines and primary cells. **A,** A panel of established AML cell lines with diverse genetic features and normal CD34^+^ bone marrow cells were treated with the indicated concentrations of CB-5339 for 72 hours. Cell viability was determined by MTT assay. *N* = 3 ± SD. IC_50_ values for each cell line are indicated. **B,** Primary AML cells from patients (*N* = 4) were treated with the indicated concentration of CB-5339 for 72 hours. Cell viability was determined by MTT assay. *N* = 3 ± SD. Characteristics of primary AML specimens are indicated. **C,** CB-5339 induces time-dependent accumulation of ubiquitin-conjugated proteins. Primary AML cells from patient #1, #2, and #3 were treated with 300 nmol/L CB-5339 for the indicated times. The effects of drug treatment on ubiquitin-conjugated protein levels were determined by immunoblotting. GAPDH documented equal protein loading. **D,** CB-5339 induces dose-dependent apoptosis. A panel of seven established AML cell lines was treated with the indicated concentrations of CB-5339 for 48 hours. Drug-induced apoptosis was quantified by PI/FACS analysis. *N* = 3 ± SD. **E,** CB-5339 triggers time-dependent DNA damage and ER stress. MOLM-13 cells were treated with increasing concentrations of CB-5339 (0–2,500 nmol/L) for 3, 5, or 12 hours. Western blotting was performed to assess the impact of drug treatment on the indicated protein levels of γ-H2AX, cleaved caspase-3, PARP, XBP1s, CHOP, and ATF-6. Arrows denote cleaved PARP and ATF6. GAPDH was used as a loading control.

We previously demonstrated that AML cells exhibit heightened sensitivity to disruption of protein homeostasis through inhibition of the NEDD8-activating enzyme with pevonedistat (27–29), providing a rationale to explore additional regulators of protein dynamics as therapeutic targets. CB-5339 is a novel, orally bioavailable p97 inhibitor currently in clinical trials with demonstrated preclinical anticancer activity (30). We therefore evaluated the antileukemic effects of CB-5339 in a panel of AML cell lines and primary patient specimens. CB-5339 treatment resulted in a dose-dependent reduction in cell viability across multiple AML cell lines and primary AML samples, with minimal toxicity demonstrated against normal CD34^+^ cells (Fig. 1A and B). Consistent with disruption of proteostasis, CB-5339 exposure led to accumulation of ubiquitin-conjugated proteins (Fig. 1C) and induction of apoptosis, as evidenced by increased sub-G_0_/G_1_ DNA content (Fig. 1D). These findings are aligned with the central role of p97 as a segregase in the ubiquitin–proteasome system (7). At the molecular level, CB-5339 induced dose- and time-dependent increases in γH2AX, along with robust activation of unfolded protein response mediators including ATF-6, CHOP, and XBP1s, culminating in cleavage of PARP and caspase-3 (Fig. 1E). The temporal pattern of early DNA damage and unfolded protein response activation followed by executioner caspase cleavage is consistent with engagement of multiple stress pathways that drive apoptotic cell death following p97 inhibition.

### Inhibition of p97 with CB-5339 synergistically enhances the anti-AML activity of DAC

**Figure 2.**
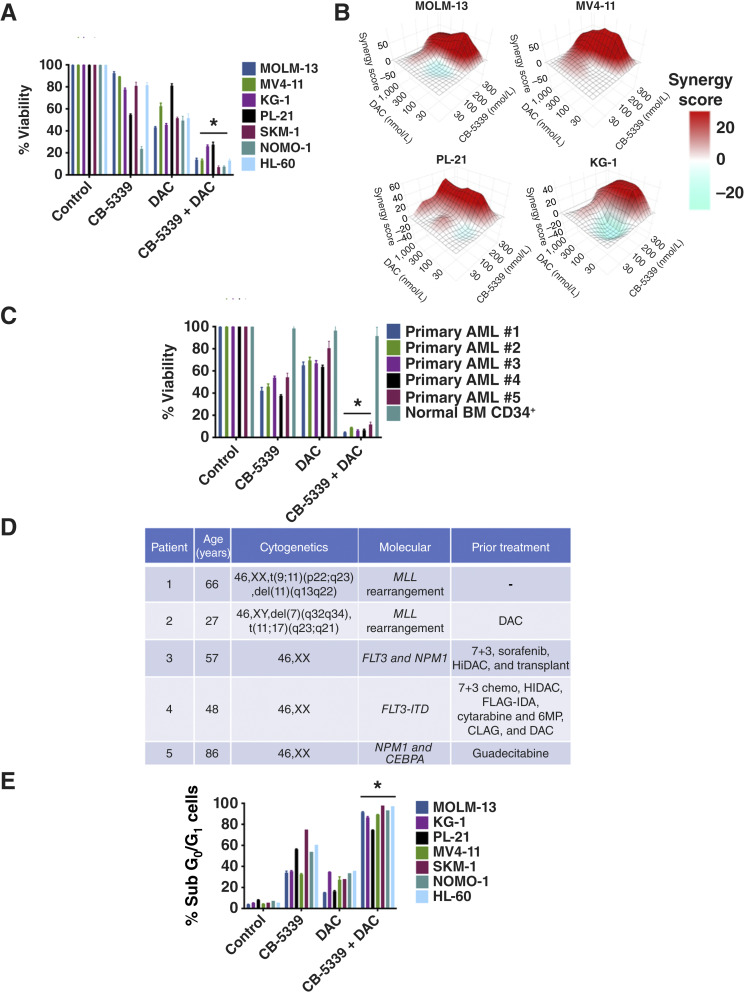
The combination of CB-5339 and DAC has synergistic antileukemic activity. **A,** CB-5339 and DAC cooperate to diminish AML cell viability in seven AML cell lines. AML cell lines were treated with 300 nmol/L DAC, 200 nmol/L CB-5339, or combinations for 72 hours. Cell viability was determined by MTT assay. *N* = 3 ± SD; * indicates significant difference from control and single agent groups, *P* < 0.005. **B,** Heatmaps of formal synergy analysis of the combination of CB-5339 and DAC in four representative AML cell lines. **C,** CB-5339 and DAC have synergistic and selective antileukemic activity against primary AML cells from patients with adverse features. Primary AML cells from patients (*N* = 5) and normal CD34^+^ bone marrow cells were treated with 500 nmol/L DAC, 250 nmol/L CB-5339, or the combination for 72 hours. Cell viability was determined by MTT assay. *N* = 3 ± SD; * indicates a significant difference from control and monotherapies, *P* < 0.005. **D,** AML patient characteristics. **E,** The CB-5339/DAC combination triggers synergistic levels of apoptosis. AML cells were treated with 250 nmol/L CB-5339, 500 nmol/L DAC, or the combination for 48 hours. Drug-induced apoptosis was quantified by PI/FACS analysis. *N* = 3 ± SD; * denotes a significant difference from control and single agents, *P* < 0.005.

We next evaluated whether p97 inhibition with CB-5339 augments the antileukemic activity of the hypomethylating agent DAC. Cell viability assays demonstrated a marked enhancement of AML cell death following combined treatment compared with either agent alone (Fig. 2A). Formal synergy analyses confirmed strong synergistic interactions across multiple AML cell lines representing diverse genetic backgrounds, including adverse-risk subtypes harboring *FLT3*-ITD, *KMT2A* rearrangements, and *TP53* mutations (Fig. 2B; Supplementary Table S1; Supplementary Fig. S1). The activity of the combination was further validated in primary patient-derived AML specimens and normal bone marrow CD34^+^ cells. Blasts obtained from patients with high-risk molecular features, including *FLT3*-ITD and *KMT2A* rearrangements, exhibited pronounced selectivity and sensitivity to CB-5339/DAC treatment *ex vivo* (Fig. 2C and D). Synergistic effects were observed at pharmacologically relevant concentrations of both agents. Consistent with these findings, cotreatment with CB-5339 and DAC induced synergistic levels of apoptosis across multiple AML models, as assessed by PI/FACS and Annexin-V/PI analysis (Fig. 2E; Supplementary Fig. S2). Hypomethylating therapy is frequently used in combination with the BCL-2 inhibitor venetoclax. Importantly, we also observed enhanced sensitivity when AML cells were treated with CB-5339 in combination with venetoclax + DAC (Supplementary Fig. S3).

### Transcriptomic analyses identify a pro–cell death signature following CB-5339 + DAC combination treatment

**Figure 3.**
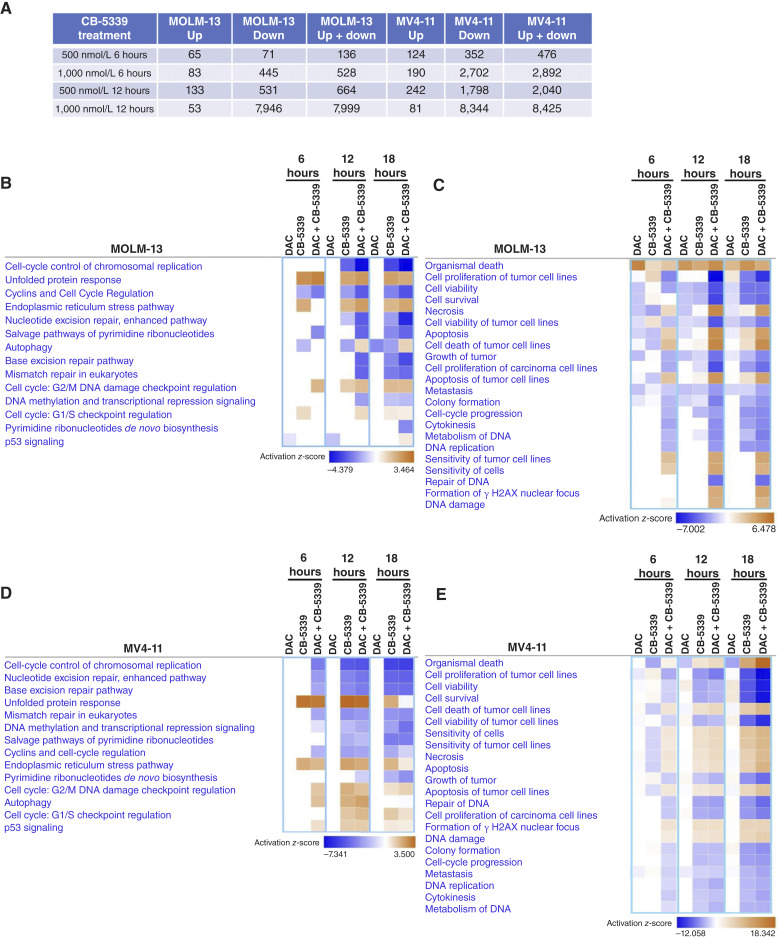
Effects of the CB-5339/DAC combination on the transcriptomic landscape. **A,** MOLM-13 and MV-4-11 cells were treated with 500 or 1,000 nmol/L CB-5339 for 6 or 12 hours. RNA sequencing was performed, and the numbers of upregulated and downregulated genes were quantified for each experimental condition. **B–E,** Ingenuity Pathway Analysis of RNA sequencing results for (**B** and **C**) MOLM-13 and (**D** and **E**) MV4-11. Cell processes and disease-related and functional pathways are sorted by absolute *Z*-score.

To define molecular correlates of synergy, we performed transcriptomic analyses in MOLM-13 and MV4-11 cells following treatment with CB-5339, DAC, or the combination at 6, 12, and 18 hours (Fig. 3A). Gene ontology enrichment analyses revealed marked induction of apoptosis-related gene programs together with pronounced suppression of DNA repair–associated pathways under combination treatment conditions (Fig. 3B–E). Among the most significantly affected pathways were those involved in homologous recombination, cell-cycle checkpoint signaling, and double-strand DNA break repair.

### CB-5339 cooperates with DAC to decrease PLK1 expression

**Figure 4.**
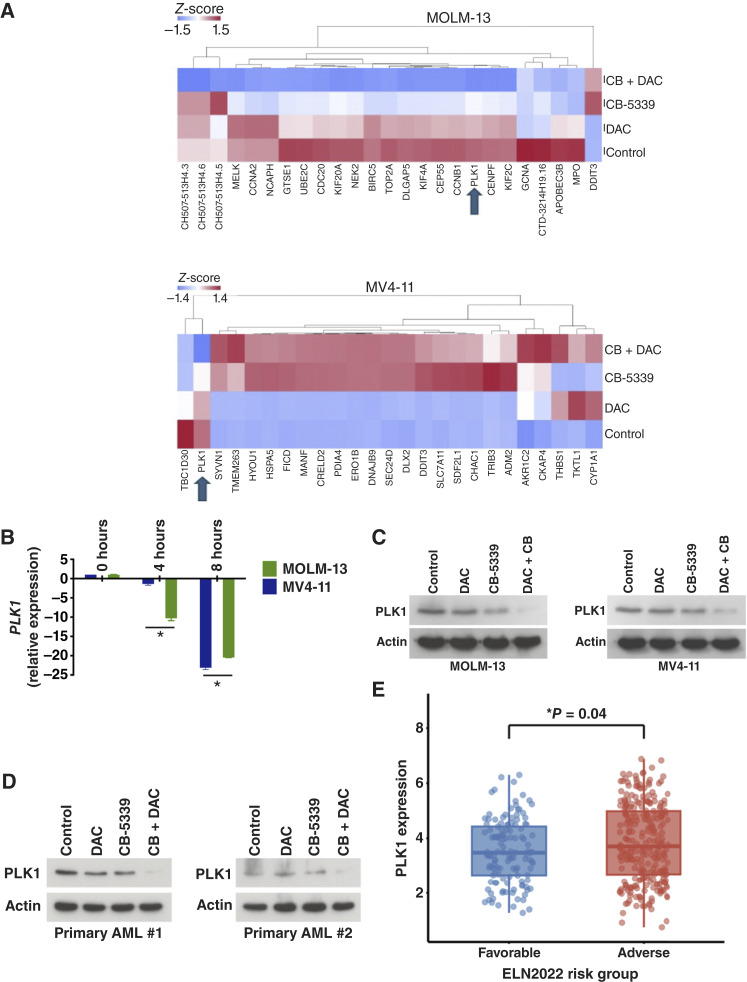
Transcriptomic analyses identify *PLK1* as a top early downregulated pharmacodynamic (PD) target of the CB-5339/DAC combination. **A,** MOLM-13 and MV4-11 cells were treated with 500 nmol/L CB-5339, 1 μmol/L DAC, or both drugs for 6 hours (MV4-11) or 12 hours (MOLM-13) and subjected to RNA sequencing analyses. Time points were selected based on the kinetics of the PD response for each individual cell line to calibrate gene level effects. Heatmaps depict the top PD targets in each cell line. **B,** qRT-PCR validates the impact of treatment with the CB-5339/DAC combination on *PLK1* expression. MV4-11 and MOLM-13 cells were treated with the combination of 250 nmol/L CB-5339, 500 nmol/L DAC for 0, 4, and 8 hours. Relative gene expression levels were quantified by TaqMan qRT-PCR and normalized to 18S rRNA. *N* = 3 ± SD; * indicates a significant difference from control, *P* < 0.005. **C** and **D,** The CB-5339/DAC combination effectively diminishes PLK1 protein levels. AML cell lines and primary AML specimens were treated with 250 nmol/L CB-5339, 500 nmol/L DAC, or both drugs for 24 hours. The impact of drug treatment on PLK1 protein levels was determined by immunoblotting. Actin documented equal protein loading. **E,***PLK1* expression is significantly upregulated in patients with AML with adverse-risk features. Patients with AML were stratified by ELN2022 risk group (favorable vs. adverse), and PLK1 expression was quantified. Each dot represents an individual patient. The center line indicates the median and boxes represent the interquartile range. Statistical significance was assessed using a two-sided Wilcoxon rank-sum test (*, *P* = 0.04).

An analysis of concordant significantly modulated genes between both MOLM-13 and MV4-11 cell lines indicated a pharmacodynamic stress-response signature as evidenced by *DDIT3* upregulation (Supplementary Fig. S4). Transcriptomic analyses identified PLK1 as one of the most significantly downregulated genes following combined CB-5339 and DAC treatment (Fig. 4A). This effect was validated at the transcript level by qRT-PCR prior to significant amounts of cell death (Fig. 4B; Supplementary Fig. S5) and at the protein level by immunoblotting in AML cell lines and primary AML blasts (Fig. 4C and D). CB-5339/DAC cotreatment produced a pronounced reduction in PLK1 expression in AML cell lines as well as in primary AML blasts (Supplementary Fig. S6). PLK1’s role at the intersection of replication recovery and mitotic progression defines a mechanistic explanation for why its downregulation under these conditions produces such pronounced cytotoxic effects (23, 31, 32). Importantly, AML patients in the adverse-risk group display significant basal upregulation of PLK1 expression (Fig. 4E).

### Silencing of *PLK1* expression enhances the anti-AML efficacy of CB-5339 and DAC

**Figure 5.**
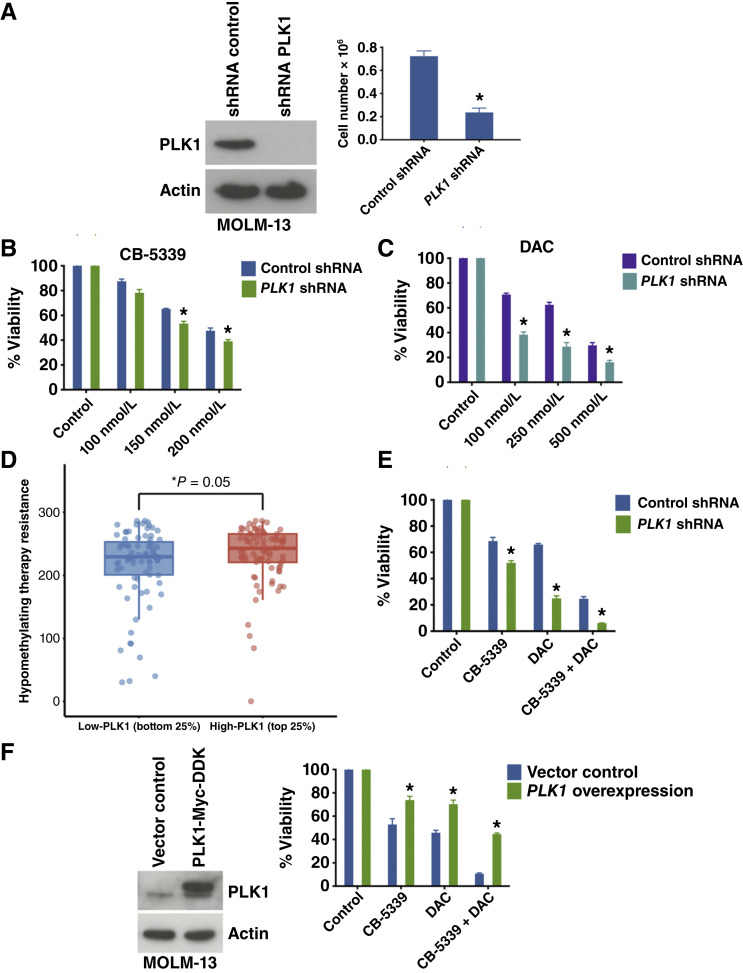
PLK1 is a key pharmacodynamic target of the CB-5339/DAC combination. **A,** Targeted *PLK1* knockdown impairs AML cell proliferation. MOLM-13 cells were transduced with nontargeted control of *PLK1*-targeted lentiviral shRNA. Knockdown efficiency was determined by immunoblotting. Cell numbers were quantified for both conditions at 48 hours after lentiviral transduction. *N* = 3 ± SD; * indicates a significant difference from control shRNA, *P* < 0.005. **B** and **C,** Effects of targeted *PLK1* knockdown on sensitivity to CB-5339 and DAC. MOLM-13 cells were infected with lentiviral nontargeted control or *PLK1*-targeted shRNA. Cells were treated with the indicated concentrations of CB-5339 and DAC. Cell viability was determined by MTT assay. *N* = 3 ± SD; * indicates a significant difference from control shRNA, *P* < 0.05. **D,** High PLK1 expression is associated with resistance to hypomethylating therapy. Within the ELN2022 adverse-risk group, patients with AML were stratified based on PLK1 levels comparing PLK1-low expression (bottom 25%) and PLK1-high expression (top 25%). Hypomethylating therapy response (azacitidine) is shown as an AUC in which higher values indicate greater resistance. Each dot represents an individual patient. Statistical significance was determined using a two-sided Wilcoxon rank-sum test (*, *P* = 0.05). **E,** PLK1 is a determinant of sensitivity to the CB-5339/DAC combination. MOLM-13 cells transduced with nontargeted control shRNA or *PLK1*-targeted shRNA lentiviral vectors were treated with 250 nmol/L CB-5339, 500 nmol/L DAC, or both drugs for 72 hours. The impact of drug treatment on cell viability was determined by MTT assay. *N* = 3 ± SD; * indicates a significant difference from control shRNA, *P* < 0.005. **F,** Overexpression of PLK1 promotes resistance to CB-5339 and DAC. PLK1 was overexpressed in MOLM-13 cells using PLK1-Myc-DDK lentiviral particles. Overexpression efficiency was assessed by immunoblotting. Vector control and PLK1-overexpressing cells were treated with 200 nmol/L CB-5339, 300 nmol/L DAC, and the combination for 72 hours. The effects of drug treatment on cell viability were quantified for each experimental condition by MTT assay. Mean ± SD, *n* = 3. * indicates a significant difference from vector control cells, *P* < 0.05.

CRISPR knockout of *PLK1* is lethal demonstrating the importance of the target for cell survival. This is consistent with complete knockout of p97 being embryonic lethal in mice (33). To functionally validate the role of PLK1 in mediating sensitivity to p97 inhibition and hypomethylating therapy, we genetically suppressed *PLK1* expression in AML cells using lentiviral shRNA. Targeted knockdown of *PLK1* significantly impaired AML cell proliferation and recapitulated the cytotoxic effects observed with pharmacologic cotreatment (Fig. 5A). Genetic suppression of *PLK1* also increased sensitivity to CB-5339 (Fig. 5B) and, to an even greater extent, to DAC (Fig. 5C). Consistent with these findings, high PLK1 expression in patients with AML was associated with resistance to hypomethylating therapy (Fig. 5D). Importantly, *PLK1* knockdown also enhanced the antileukemic activity of combined CB-5339 and DAC treatment (Fig. 5E). To further evaluate the relationship between PLK1 levels and sensitivity to CB-5339 and DAC, we overexpressed *PLK1* in MOLM-13 cells using a lentiviral PLK1-Myc-DDK construct. Consistent with our knockdown data, forced overexpression of *PLK1* significantly blunted cell death induced by CB-5339, DAC, and the combination (Fig. 5F). Collectively, these data support an important role of PLK1 in regulating therapeutic response to CB-5339 and DAC.

### The combination of CB-5339 and DAC is well tolerated and extends survival in an orthotopic AML xenograft model

**Figure 6.**
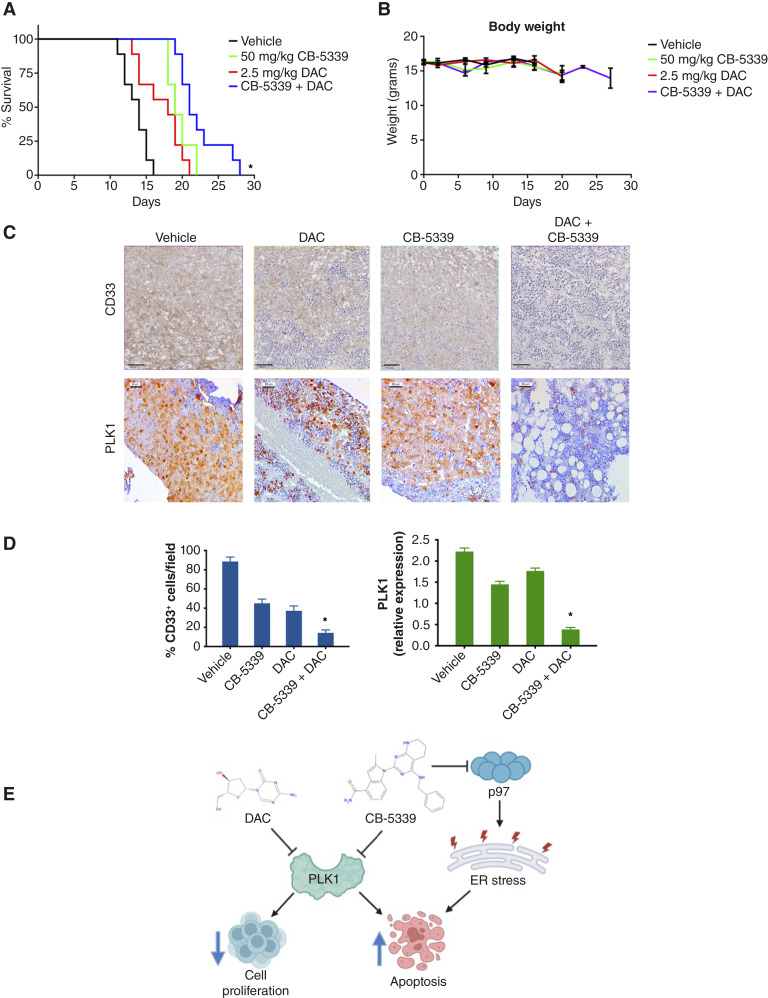
CB-5339 in combination with DAC prolongs survival and is well tolerated in an orthotopic AML xenograft model. **A,** The CB-5339/DAC combination significantly increases overall survival. A disseminated mouse model of AML was generated by injecting MV4-11 cells into the tail veins of NOG mice. Mice were treated with vehicle control, 50 mg/kg CB-5339 daily (oral), 2.5 mg/kg DAC twice weekly (intraperitoneally), or both drugs for the duration of the study. Overall survival for each group was determined by Kaplan–Meier analysis. *N* = 10 ± SD; * indicates a significant difference from control and single-agent groups, *P* < 0.005. Median survival values are as follows: vehicle = day 14, CB-5339 = day 19, DAC = day 18, and CB-5339 + DAC = day 22. **B,** The CB-5339/DAC combination is well tolerated. Mouse weight is shown for all treatment groups. *N* = 10 ± SD. **C,** Administration of the CB-5339/DAC combination diminishes CD33^+^ blasts and PLK1 levels *in vivo*. Bone marrow specimens were collected from mice in all experimental groups and subjected to IHC analysis to quantify PLK1 and CD33 (leukemia cell marker) levels. Scale bar, 50 μm. **D,** Quantification of IHC analyses. The number of CD33^+^ cells were counted in five random fields. For PLK1, signal intensity was quantified using ImageJ software. *N* = 5 ± SD; * indicates a significant difference from control and single-agent groups, *P* < 0.005. **E,** Schematic illustration of the pharmacodynamic synergy of the CB-5339/DAC combination.

We next evaluated the antileukemic activity of CB-5339 and DAC *in vivo* using a disseminated AML xenograft model. Treatment with the combination significantly prolonged overall survival compared with vehicle control and either single agent alone (Fig. 6A). The regimen was well tolerated, with no significant weight loss or overt signs of toxicity observed over the course of treatment (Fig. 6B). *Ex vivo* analysis of bone marrow specimens from treated mice demonstrated a marked reduction in CD33^+^ leukemic blasts together with suppression of PLK1 protein expression (Fig. 6C and D).

## Discussion

Inhibition of p97 compromises extraction and processing of misfolded and regulatory protein substrates, amplifies ER stress, and interferes with resolution of aberrant replication intermediates (34–36). DAC, by incorporating into DNA and depleting DNMT1, imposes replication stress and disrupts DNA methylation-coupled repair processes (10). When combined, these pressures overwhelm checkpoint capacity in AML cells. In this stressed context, PLK1 becomes indispensable for survival, yet its levels are simultaneously diminished under p97 blockade. This two-hit failure of stress adaptation and cell-cycle control converts PLK1 from a resistance factor into an Achilles’ heel that drives potent antileukemic synergy (Fig. 6E).

Whereas direct inhibition of PLK1 has long been pursued as a therapeutic strategy in AML, clinical experience has underscored the difficulty of targeting a core mitotic kinase without incurring unacceptable toxicity (37–40). The limited efficacy and tolerability of first-generation PLK1 inhibitors reflect a broader challenge in AML drug development as essential cell-cycle regulators rarely offer a sufficient therapeutic window when targeted directly. Our findings demonstrate that functional suppression of PLK1 achieved by destabilizing the cellular state that necessitates its activity provides a more effective and potentially tolerable alternative. By collapsing the conditions that require PLK1 while simultaneously reducing PLK1 abundance, the CB-5339/DAC combination circumvents limitations that have historically constrained PLK1-directed therapies. This stress-contingent dependency defines a mechanistic basis for the robust synergy observed across genetically diverse AML models.

Concurrent p97 inhibition disables resolution of proteotoxic and replication-associated damage, dismantling compensatory stress-adaptation programs. Under these conditions, reduced PLK1 expression becomes particularly deleterious, converting a survival requirement into a liability. Consistent with this model, the CB-5339/DAC combination retains activity across adverse-risk genotypes, including *FLT3-ITD*, *KMT2A* rearrangements, and *TP53* mutations, indicating that it exploits a shared vulnerability rather than a mutation-specific dependency. Targeting fundamental stress-resolution pathways may therefore impose selective pressure that AML cells are less able to evade (41).

From a translational perspective, the tolerability of the CB-5339/DAC regimen *in vivo* strengthens the clinical relevance of this approach. The absence of overt toxicity, together with pharmacodynamic suppression of PLK1 in leukemic blasts, indicates that functional targeting of PLK1 through p97 inhibition preserves antileukemic efficacy while avoiding the dose-limiting toxicities arising from sustained, global blockade of mitotic progression associated with direct PLK1 inhibitors. The CB-5339/DAC combination induces context-dependent suppression of PLK1 within a broader stress-response framework, which may provide a more favorable therapeutic window. A comprehensive evaluation of hematologic toxicity, including effects on normal hematopoiesis, will be important in future preclinical studies and clinical development. This consideration is particularly important for elderly patients and those with comorbidities, for whom hypomethylating agents remain the therapeutic backbone (42). Future studies will also investigate the CB-5339/DAC combination in patient-derived xenografts and additional primary patient specimens to identify potential vulnerabilities in specific AML subtypes. Several features make this strategy immediately actionable. It combines a second-generation p97 inhibitor (CB-5339) already in clinical development with an established hypomethylating backbone, enabling near-term translation without additional formulation barriers. Synergy occurs at pharmacologically relevant doses, and pharmacodynamic biomarkers, including PLK1 expression, γH2AX induction, and apoptotic readouts, are readily measurable. These markers can be integrated into early-phase clinical trials to confirm mechanism engagement, guide dose and schedule selection, and link pharmacodynamic effects to clinical endpoints such as composite complete remission and duration of response. CB-5339 was originally developed by Cleave Therapeutics and is now being further advanced into clinical testing by Casi Pharmaceuticals.

In summary, this study establishes p97 inhibition as a powerful means to exploit stress-adaptation dependencies in AML and identifies functional suppression of PLK1 as a central mechanism underlying synergy with hypomethylating therapy. By shifting the therapeutic paradigm from direct kinase inhibition to stress-mediated vulnerability, these findings provide a strong rationale for clinical evaluation of CB-5339 in combination with DAC. More broadly, they support targeting adaptive stress responses as a rational strategy to overcome resistance in genetically complex myeloid malignancies.

## Supplementary Material

Supplemental Table S1 Table S1. Combination index (CI) values of AML cells treated with CB-5339 and DAC.

Supplemental Figure S1 Figure S1. 2D Synergy heatmaps of the CB-5339/DAC combination.

Supplemental Figure S2 Figure S2. CB-5339 and DAC synergize to trigger apoptosis.

Supplemental Figure S3 Figure S3. CB-5339 synergizes with venetoclax (VEN) and DAC to reduce AML cell viability.

Supplemental Figure S4 Figure S4. Gene modulation overlap between MOLM-13 and MV4-11 cells.

Supplemental Figure S5 Figure S5. Time course cell viability analysis following treatment with CB-5339, DAC, and the combination.

Supplemental Figure S6 Figure S6. Quantification of PLK1 immunoblotting from (A) Figure 4C and (B) 4D. Band intensity was measured using Image J software.

## Data Availability

The data generated in this study are available within the main article and its Supplementary Data files. Sequencing data have been uploaded to the NCBI Sequence Read Archive (https://www.ncbi.nlm.nih.gov/sra) database under data repository BioProject accession number PRJNA1469647. Data are also available upon reasonable request to the corresponding author.
