# Supplementary figures and images for: p97 Inhibition Synergistically Enhances Hypomethylating Therapy through Targeting of PLK1 in Acute Myeloid Leukemia
Source: Cancer Res Commun. 2026 Jun 26;6(6):1509–21. doi: 10.1158/2767-9764.CRC-26-0035 (PMC13306721; doi:10.1158/2767-9764.CRC-26-0035)

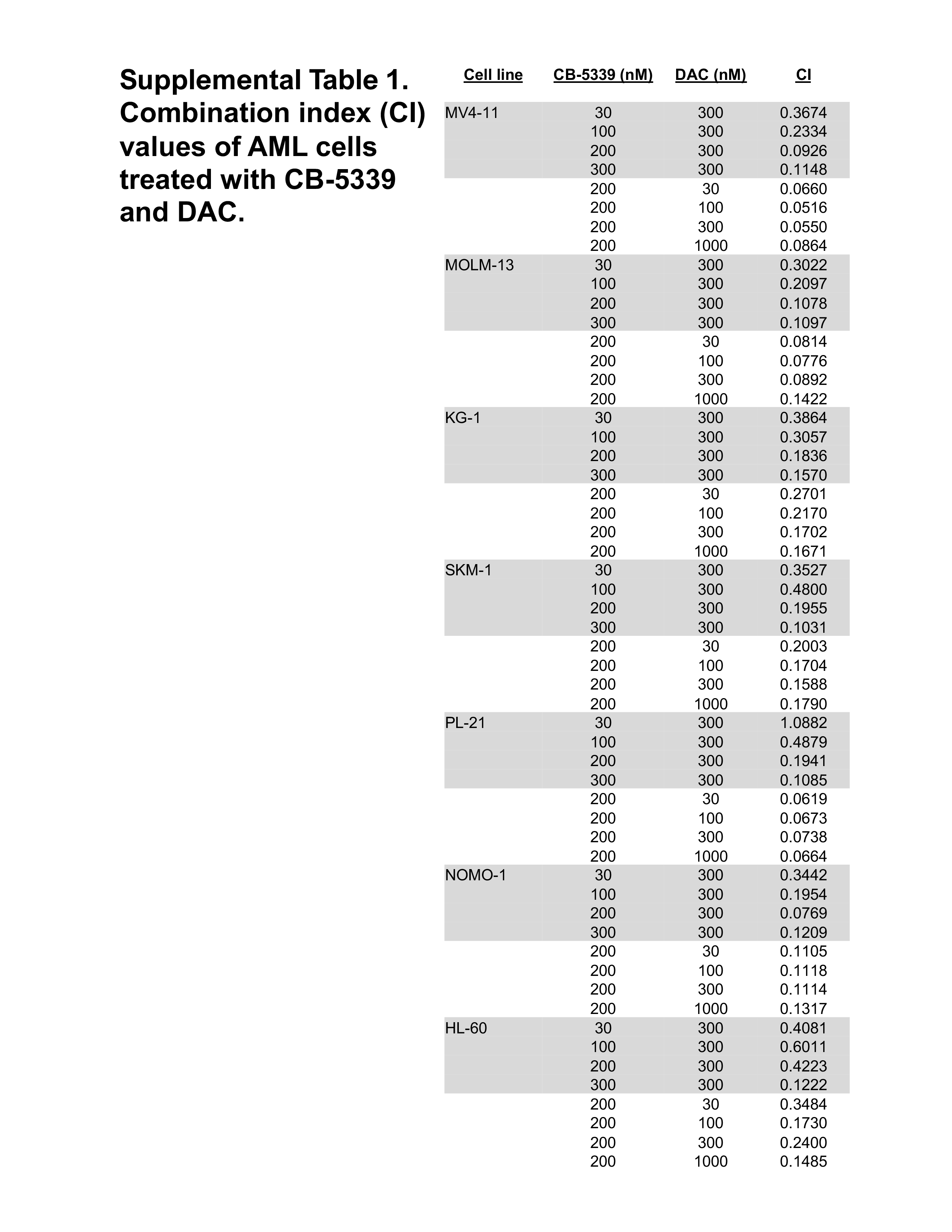

Supplement: Supplemental Table S1 — Table S1. Combination index (CI) values of AML cells treated with CB-5339 and DAC. [file crc-26-0035_supplemental_table_s1_suppst1.png]

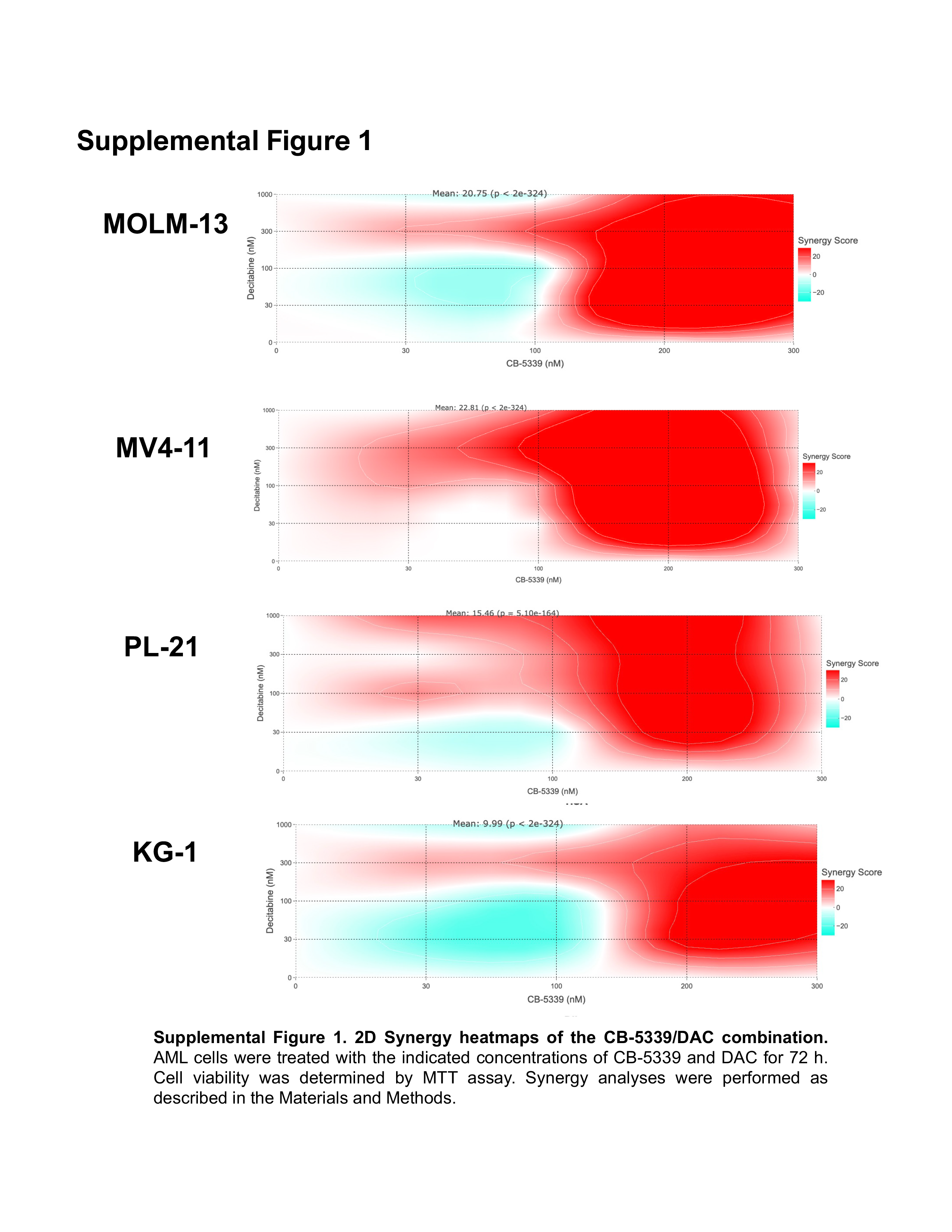

Supplement: Supplemental Figure S1 — Figure S1. 2D Synergy heatmaps of the CB-5339/DAC combination. [file crc-26-0035_supplemental_figure_s1_suppsf1.png]

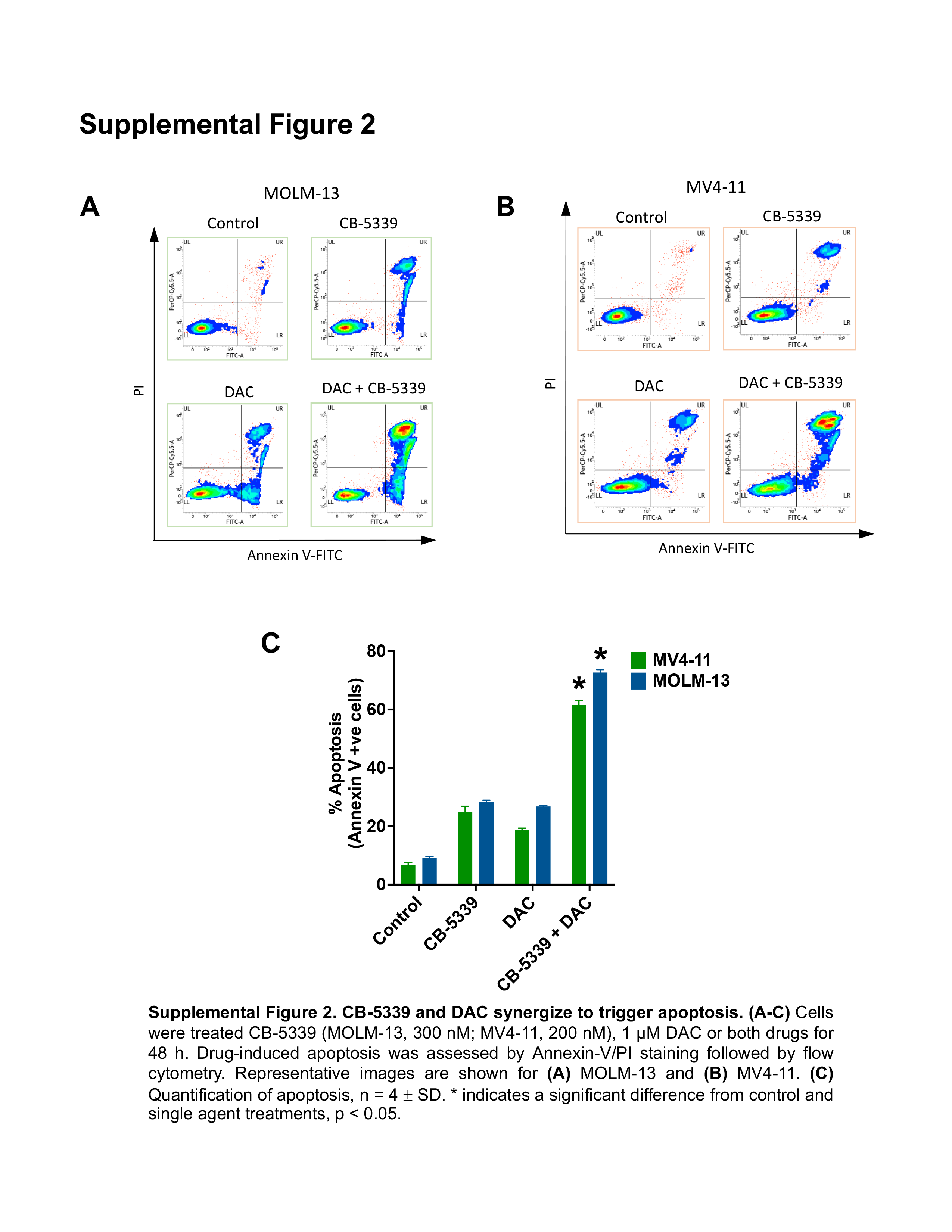

Supplement: Supplemental Figure S2 — Figure S2. CB-5339 and DAC synergize to trigger apoptosis. [file crc-26-0035_supplemental_figure_s2_suppsf2.png]

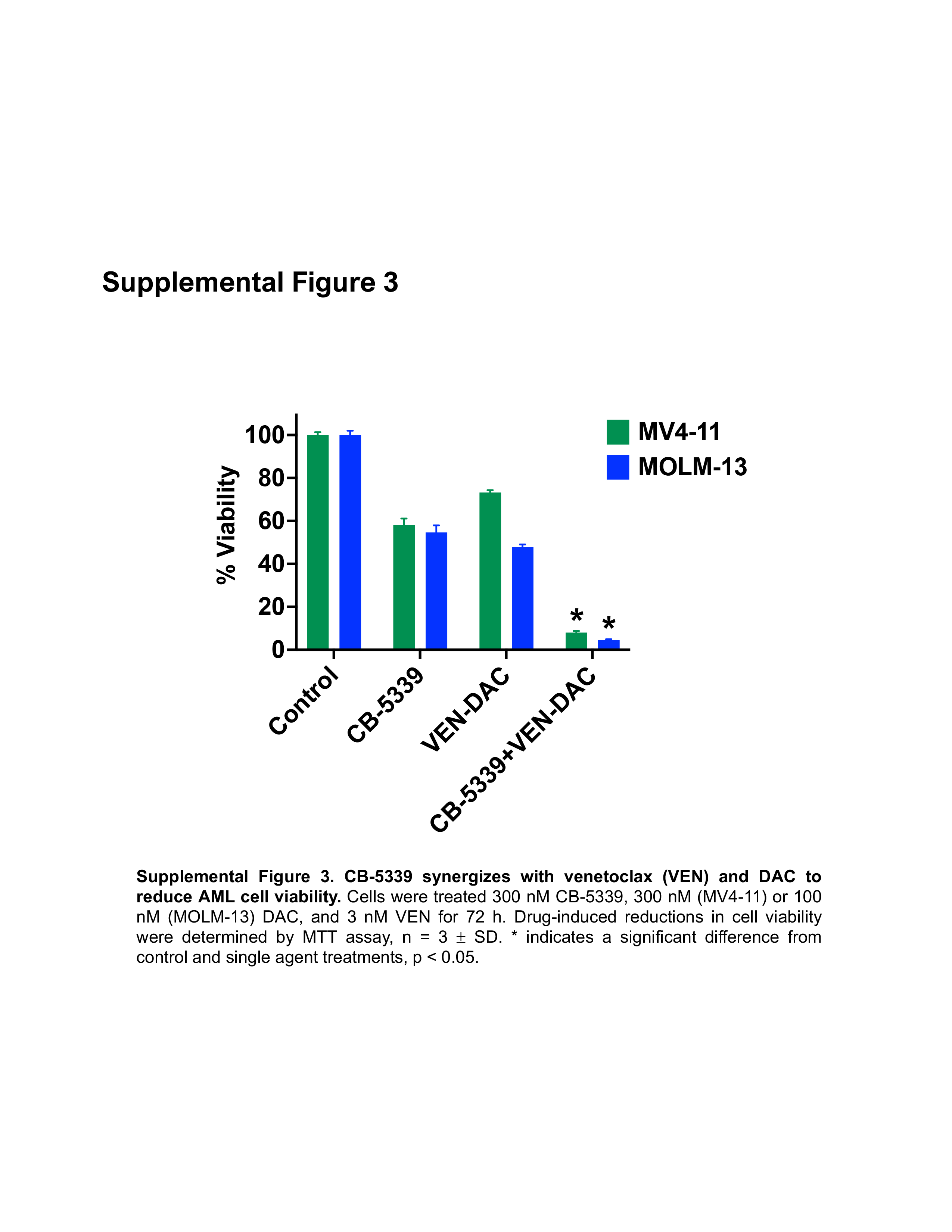

Supplement: Supplemental Figure S3 — Figure S3. CB-5339 synergizes with venetoclax (VEN) and DAC to reduce AML cell viability. [file crc-26-0035_supplemental_figure_s3_suppsf3.png]

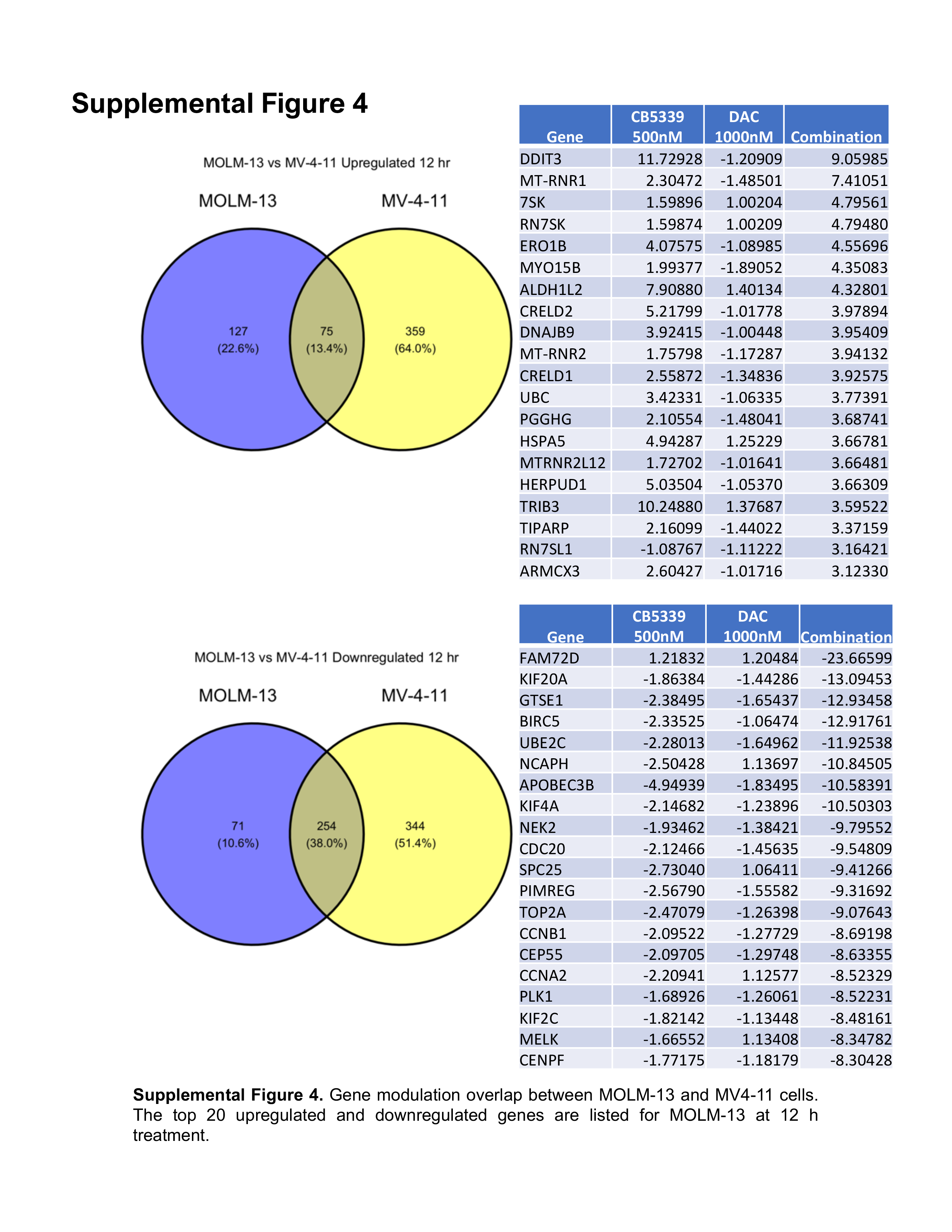

Supplement: Supplemental Figure S4 — Figure S4. Gene modulation overlap between MOLM-13 and MV4-11 cells. [file crc-26-0035_supplemental_figure_s4_suppsf4.png]

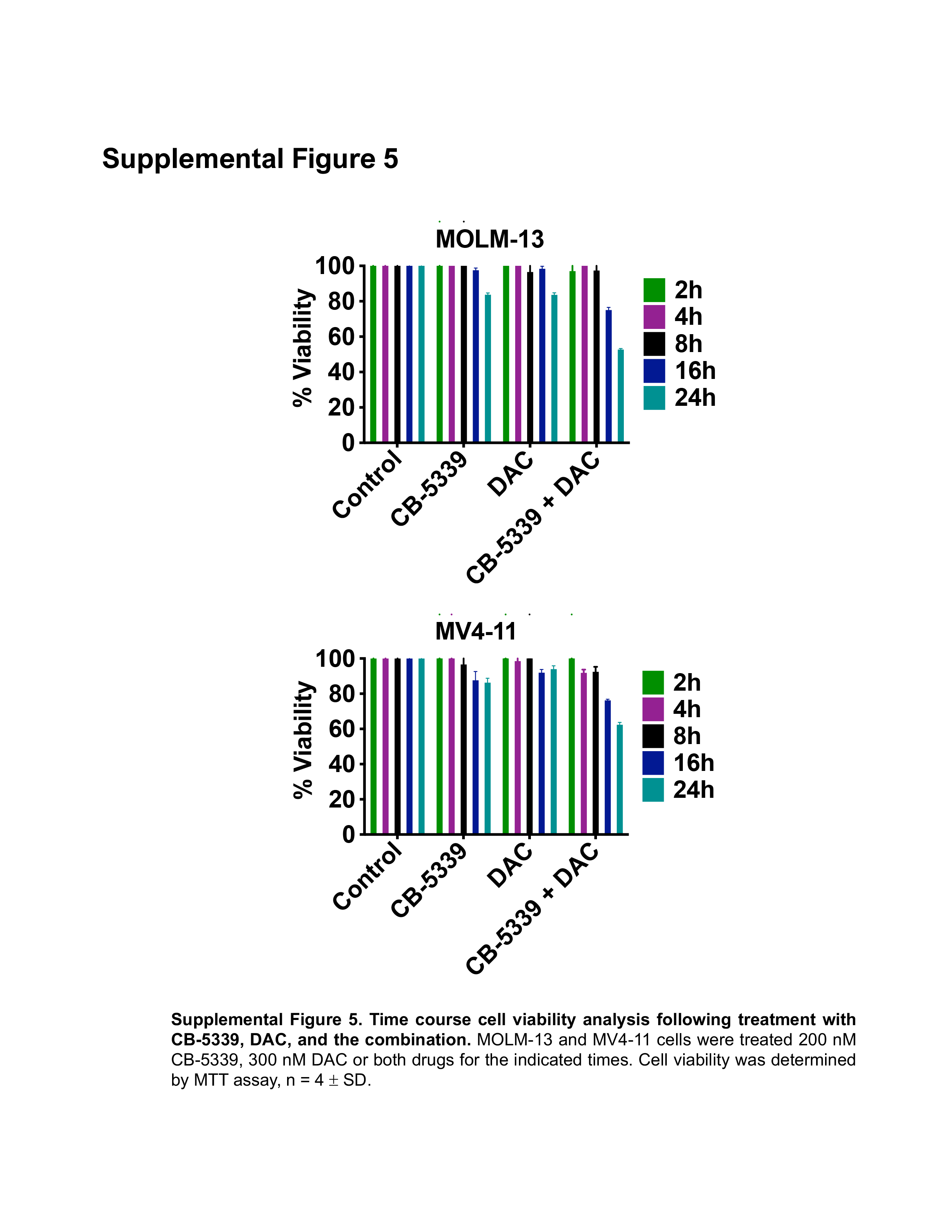

Supplement: Supplemental Figure S5 — Figure S5. Time course cell viability analysis following treatment with CB-5339, DAC, and the combination. [file crc-26-0035_supplemental_figure_s5_suppsf5.png]

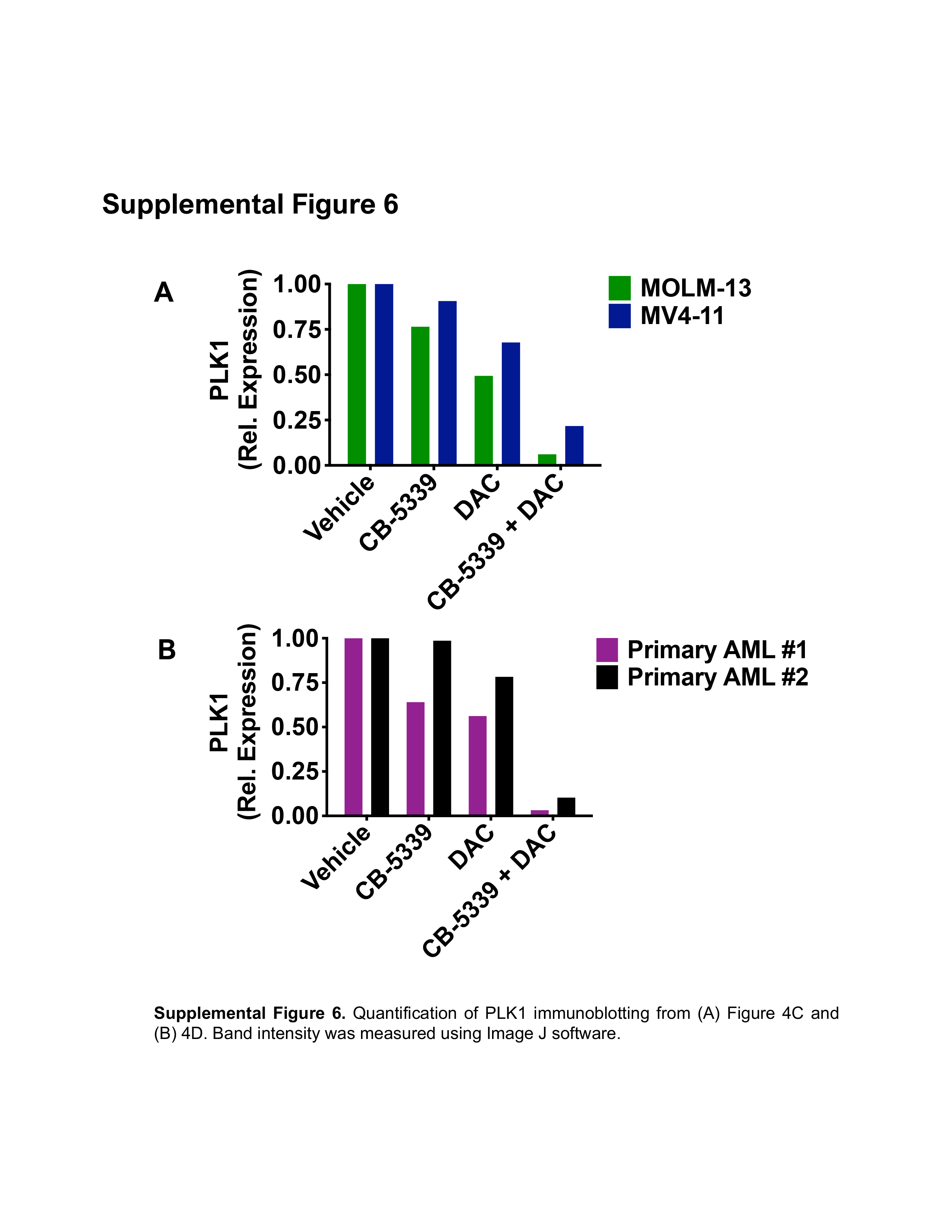

Supplement: Supplemental Figure S6 — Figure S6. Quantification of PLK1 immunoblotting from (A) Figure 4C and (B) 4D. Band intensity was measured using Image J software. [file crc-26-0035_supplemental_figure_s6_suppsf6.png]
